# Dichotomous roles of neutrophils in modulating pathogenic and repair processes of inflammatory bowel diseases

**DOI:** 10.1093/pcmedi/pbab025

**Published:** 2021-11-15

**Authors:** Huimin Chen, Xiaohan Wu, Chunjin Xu, Jian Lin, Zhanju Liu

**Affiliations:** Center for Inflammatory Bowel Disease Research, the Shanghai Tenth People's Hospital, Tongji University School of Medicine, Shanghai 200072, China; Center for Inflammatory Bowel Disease Research, the Shanghai Tenth People's Hospital, Tongji University School of Medicine, Shanghai 200072, China; Department of Gastroenterology, the First People's Hospital of Shangqiu City Affiliated to Xinxiang Medical University, Shangqiu 476100, China; Department of Gastroenterology, Affiliated Hospital of Putian University, Putian 351106, China; Center for Inflammatory Bowel Disease Research, the Shanghai Tenth People's Hospital, Tongji University School of Medicine, Shanghai 200072, China

**Keywords:** neutrophil, innate immune cell, inflammatory bowel disease, intestinal inflammation, mucosal homeostasis, immune response, microbial dysbiosis

## Abstract

Neutrophils are considered as complex innate immune cells and play a critical role in maintaining intestinal mucosal homeostasis. They exert robust pro-inflammatory effects and recruit other immune cells in the acute phase of pathogen infection and intestinal inflammation, but paradoxically, they also limit exogenous microbial invasion and facilitate mucosal restoration. Hyperactivation or dysfunction of neutrophils results in abnormal immune responses, leading to multiple autoimmune and inflammatory diseases including systemic lupus erythematosus, rheumatoid arthritis, and inflammatory bowel diseases (IBD). As a refractory intestinal inflammatory disease, the pathogenesis and progression of IBD are associated with complicated immune response processes in which neutrophils are profoundly involved. However, the consensus on potential roles of neutrophils in modulating pathogenic and repair processes of IBD remains not fully understood. Accumulated infiltrating neutrophils cross the epithelial barrier and contribute to microbial dysbiosis, aggravated intestinal architectural damage, compromised resolution of intestinal inflammation and increased risk of thrombosis during IBD. Paradoxically, activated neutrophils are also associated with effective elimination of invaded microbiota, promoted angiogenesis and tissue restoration of gut mucosa in IBD. Here, we discuss the beneficial and detrimental roles of neutrophils in the onset and resolution of intestinal mucosal inflammation, hoping to provide a precise overview of neutrophil functions in the pathogenesis of IBD.

## Introduction

Neutrophils are the most abundant innate immune cells in the circulation and are capable of promptly accumulating in large numbers and responding to multiple pathogenic signals at the sites of tissue damage.^1^ Neutrophils have long been considered as an initiator in acute and chronic inflammation that are associated with excessive immune responses and aggravate tissue injuries. They are armed with effective immunoregulatory and bactericidal properties using manifold intracellular and extracellular mechanisms, including reactive oxygen species (ROS), degranulation, phagocytosis, neutrophil extracellular traps (NETs), recruitment and activation of other immune cells (e.g. macrophages, dendritic cells, natural killer cells, T and B cells).^2,3^ Despite pro-inflammatory roles, neutrophils also play an essential role in protecting the host from exogenous pathogen invasion and avoiding detrimental effects mediated by dead or injured cells.^4^

Inflammatory bowel disease (IBD), encompassing Crohn's disease (CD) and ulcerative colitis (UC), is chronic and idiopathic inflammatory diseases of unknown etiology affecting the gastrointestinal tract with increasing global prevalence.^5–7^ IBD is progressive and destructive, with heterogeneous complications including abscesses, stenoses, fistulas, extraintestinal manifestations and colitis-associated cancer, and induced by interacting genetical, environmental, microbial and immune pathogenic factors.^8,9^ Several lines of evidence have shown that intestinal mucosal T helper (Th)-related pro-inflammatory cytokines including interferon (IFN)-γ and tumor necrosis factor (TNF)-α, as well as Th17-derived cytokines including interleukin (IL)-17A and IL-23, are associated with the pathogenesis of IBD.^10–13^ Numerous studies have also demonstrated that neutrophils participate in the pathogenesis and progression of IBD. Exacerbated intestinal inflammation and mucosal damage in the early phase of IBD are associated with activated neutrophil infiltration that further contributes to the recruitment of subsequently activated immune cells.^4^ However, these accumulated neutrophils in the inflamed mucosa also phagocytose pathogenic bacteria and dysfunctional cells, and promote mucosal restoration and resolution of inflammation. The diversified functions of neutrophils endow them with a fickle role in intestinal inflammation. In this review, we summarize the dichotomous roles of neutrophils in modulating the pathogenic and repair processes of IBD and highlight their potential therapeutic application for the management of IBD.

## Physiological functions of neutrophils

Neutrophils are predominant innate immune cells with unique physiological characteristics that furnish effective immunoregulatory properties despite a short life span. As the most abundant circulated leukocytes, neutrophils can quickly migrate and recruit into the inflamed tissue, and collaborate to build the first-line defense against exogenous pathogens.^14,15^ Under intestinal homeostasis conditions, multiple functions of neutrophils including production of ROS, phagocytosis, degranulation and release of NETs enable them to modulate innate and adaptive immune responses (Table 1). When neutrophils encounter invading pathogens, ROS are formed in respiratory burst by nicotinamide adenine dinucleotide phosphate (NADPH) oxidase that effectively destroy pathogens, followed by phagocytosis to minimize deleterious effects mediated by dying or damaged cells.^4,16^ Another bactericidal weapon of neutrophils is the affluent pool of membrane and intracellular granules that include azurophilic (primary) granules containing myeloperoxidase (MPO) and defensins, specific (secondary) granules containing cathelicidin, gelatinase (tertiary) granules containing matrix metalloproteinase-9 (MMP-9), and other secretory granules containing integrins (Table 2).^17,18^

**Table 1.** The arsenal of neutrophils.

Site	Item	Components	Functions	References
Intracellular	ROS	Hydrogen peroxide (H_2_O_2_), hypochlorite ion (OCl^−^), and superoxide anion (O_2_^−^)	Kill the pathogens; promote neutrophil apoptosis	^2^
	Degranulation	Azurophilic (primary) granules: MPO, elastase, lysozyme, defensins, azurocidin; specific (secondary) granules: lactoferrin, cathelicidin; gelatinase (tertiary) granules: MMP-9; other secretory granules: integrins	Amplify inflammatory responses; recruit neutrophils; eliminate microbiota; facilitate cell adhesion and angiogenesis	^103,104^
	Phagocytosis	Receptor-mediated processes: pattern-recognition receptors; FcγRs; complement receptors	Phagocytize pathogens and cell debris	^104,105^
Extracellular	NETs	Chromatin coated with histones, proteases, granular and cytosolic bactericidal proteins	Limit and kill bacterial, fungi, viruses and parasites; promote thrombosis	^3,106^
	Cytokines	IFN-γ, TNF, L-1β, IL-4, IL-8, IL-10 and IL-12	Pro- and anti-inflammatory	^22,107^
	Chemokines	CXCL-1-6,8-13,16; CCL-2-4,17-20	Recruit both innate and adaptive immune cells (neutrophils, monocytes, macrophages, DCs, NK cells and T cells); amplify the inflammatory immune responses	^20,108–113^

ROS, reactive oxygen species; MPO, myeloperoxidase; MMP-9, matrix metalloproteinase-9; FcγRs, Fcγ receptors; NETs, neutrophil extracellular traps.

**Table 2.** Granules of neutrophils.

Granule type	Granules	Functions	References
Primary or azurophilic granules	MPO, elastase, lysozyme, defensins, azurocidin, BPI, cathepsin G, proteinase 3, sialidase, β-glucuronidase	Oxidative burst; antimicrobial effect; proteolytic effect; recruitment and activation of other immune cells	^17,18,114^
Secondary or specific granules	Lysozyme, lactoferrin, NGAL, hCAP-18, β2-microglobulin, collagenase, pentraxin 3	Antimicrobial effect; proteolytic effect; modulation and suppression inflammatory response	^17,18,115^
Tertiary or gelatinase granules	Gelatinase, MMP-8, MMP-9, MMP-25, arginase-1, leukolysin	Antimicrobial effect; tissue restoration; mobilization of cytokines	^17,18,93,99^
Secretory vesicles	FcγRIII, fMLP receptors, β2 integrins, complement receptor 1, plasma proteins	Neutrophil chemoattraction; cell adhesion	^17,18^
Membrane granules	NOX2 (Gp91phox/p22phox), Mac-1 (CD11b/CD18), CD10, CD14, CD35, CD63, CD68, CD177, G-CSFR	Host defense against invaded microbes; neutrophil migration	^16–18,87,116,117^
Other proteins	Alkaline phosphatase, histones, calprotectin	Toxicity to invaded pathogens; regulation of cell division; regression of inflammation	^17,18,118,119^

BPI, bactericidal/permeability-increasing protein; NGAL, neutrophil gelatinase-associated lipocalin; hCAP-18, human 18 kDa cathelicidin antimicrobial protein; NOX2, NADPH oxidases 2; Mac-1, Macrophage 1 antigen; G-CSFR, granulocyte colony-stimulating factor receptor.

Neutrophils are capable of forming and releasing NETs which can degrade and eradicate pathogens, and prevent them from uncontrolled spreading.^19^ Once neutrophils arrive at the inflamed tissues, they can recruit themselves (through CXCL-1, -2, -5 and -8) and other immune cells including monocytes (through CCL-2, -3 and -4), macrophages (through CCL-2, -3 and -4), NK cells (through CCL-2, -3, -4 and CXCL-10), dendritic cells (through CCL-2, -4, -18, -19 and -20), and T cells (through CXCL-9, -10, -11, -12, CCL-2, -17, -18 and -20).^20^ IL-8 produced by the intestinal epithelial cells (IECs) also triggers neutrophil recruitment to the intestinal lamina propria, thus further proving the interaction between neutrophils and other immune cells.^21^ Additionally, neutrophils secrete several pro- and anti-inflammatory cytokines including IFN-γ, TNF, IL-4, and IL-10 in response to inflammatory signals and pathogens associated molecular patterns (PAMPs).^22^ Taken together, neutrophils not only participate in the occurrence and development of inflammation, but also facilitate resolution of inflammation and healing of damaged tissue, and ultimately maintain intestinal immune homeostasis.

## Recruitment and infiltration of neutrophils in inflamed mucosa of IBD

Although the precise etiology and pathology of IBD remain unclear, multifactorial pathological injury (e.g. compromised epithelial barrier integrity) and dysregulated innate and adaptive immune responses are proved to be associated with the pathogenesis of IBD.^23,24^ Massive recruitment and accumulation of neutrophils are observed in the intestinal mucosa of IBD, accompanied with upregulated levels of protein arginine deiminase 4, elastase, MPO and NETs in the colonic mucosa of UC patients, even in clinical remission.^4,25,26^

Recruitment and infiltration of neutrophil into inflamed mucosa of IBD are in a multifactor-dependent manner. Increased cytokines (e.g. IL-1β, IL-6, IL-8, IL-17, TNF-α, GM-CSF and G-CSF) and chemokines (e.g. CXCL-1, -8 and -10) produced by neutrophils themselves and other immune cells (e.g. IECs, macrophages and fibroblasts) during intestinal inflammation are important contributors to recruitment and infiltration of neutrophils into the intestinal mucosa.^27–30^ The complement component C5a is an important chemoattractant of neutrophils, while decreased neutrophil infiltration is found in the colon of *C5ar*^−/−^ mice and anti-C5a-treated wild-type mice in dextran sulphate sodium (DSS)- or 2,4,6-trinitronitrobenzene sulfonic acid (TNBS)-induced colitis.^31,32^ Additionally, an increase of leukotriene B_4_ and hepoxilin A_3_ also promotes neutrophil infiltration in IBD.^33,34^ Moreover, neutrophils can be recruited by bacteria-derived metabolites, such as formyl-methionylleucyl-phenylalanine (fMLF) and short-chain fatty acids (e.g. acetate and butyrate).^35–37^ Upregulated IEC-derived MMPs (e.g. MMP-3 and MMP-7) result in enhanced migration of neutrophils by regulating activity of chemokines (e.g. CXCL-7 and CXCL-8) during intestinal inflammation.^38,39^ Intriguingly, monocyte chemotactic protein 1-induced protein 1 (MCPIP-1) is found to be highly expressed in neutrophils of IBD patients and profoundly suppresses neutrophil migration and neutrophil-mediated pro-inflammatory responses via a MCPIP-1-mediated negative feed-back loop.^40^ However, neutrophils are also observed to evolve an intrinsic mechanism that restrains uncontrolled aggregation through G protein-coupled receptor (GPCR)-mediated desensitization, which further allows neutrophils to scan wider tissue areas for pathogens during inflammation.^41^ Thus, despite many neutrophil chemoattractants released by themselves, neutrophils can also self-limit their migration and infiltration at inflammatory state and maintain the homeostatic regulation in gut mucosa.

During intestinal inflammation, activated neutrophils undergo transendothelial migration (including tethering, rolling, adhesion, crawling and transmigration) and transepithelial migration to reach the inflamed mucosa in the stimulation of multiple chemoattractant gradients within minutes.^42,43^ Under physiological conditions, apoptotic neutrophils in the intestinal mucosa are phagocytosed by macrophages through efferocytosis which prevents neutrophil lysis and dampens immune responses.^44^ However, under inflammatory or infectious conditions, the longevity of neutrophils can be extended by a variety of inflammatory mediators such as G-CSF, GM-CSF, component C5a, C-reactive protein (CRP), serum amyloid A (SAA), bacterial components, and pro-inflammatory cytokines (e.g. IL-1β, IL-6, IL-8 and IFN-γ). The apoptosis of neutrophils can then be delayed which allows neutrophils to carry out more durable activities and accumulate in the gut mucosa.^45–49^ Accordingly, orderly recruitment, activation and apoptosis of neutrophils are indispensable for resolution of intestinal inflammation, while dysfunction in any of these procedures leads to aggravating mucosal inflammation and intestinal damage consequently.

## Pathogenic roles of neutrophils in the pathogenesis of IBD

Under conditions of gut inflammation, neutrophils migrate from the circulation toward the intestine mucosa and result in a robust inflammatory response, characterized by destruction of the mucosal epithelial barrier, dysregulated immune responses to commensal bacteria, and defects in the resolution of the intestinal inflammation.

### Neutrophils amplify immune responses and exacerbate intestinal mucosal damage

Given that neutrophils are capable of recruiting and activating plentiful immune cells to participate in the intestinal inflammation, conspiracy of these immune cells would bring about inflammatory cascades and epithelial damages that are associated with the development of IBD.^50^ As a prominent hallmark of IBD, dysregulated immune response to commensal bacteria contributes to the compromised epithelial barrier integrity and chronic relapsing intestinal inflammation.^51,52^ Neutrophils equipped with manifold antimicrobial weapons exert potent bactericidal functions to limit the dissemination of deleterious bacteria, but inevitably cause tissue damage by excessive production of ROS, MPO, MMPs, pro-inflammatory cytokines and NETs. Indeed, proteases (e.g. proteinase 3, elastase and MMPs) released by transepithelial neutrophils weaken the mucosal barrier through disrupting adherens junctions and guiding neutrophils into a direction of basolateral to apical, as demonstrated in T84 (one of human intestinal epithelial cells) monolayers.^53^ Additionally, NETs are demonstrated to contribute to the pathogenesis of intestinal inflammation through the impairment of mucosal barrier function in mouse colitis models induced by DSS or TNBS. NETs compromise gut mucosal permeability, induce the apoptosis of IECs, and destroy the integrity of adherens junctions and tight junctions which promotes luminal microbial dissemination and initiation of mucosal inflammation.^54^ Consistently, experimental colitis models also prove that depletion of neutrophils using neutralizing antibodies has been shown to ameliorate DSS/TNBS-induced colitis in rat and in mice.^55,56^ In patients with IBD, massive amounts of neutrophils migrate to colonic epithelial crypts and form cryptitis and crypt abscesses, which further cause damage of the physiological architecture of crypt, leading to intestinal mucosa injury.^42^

Moreover, immunoglobulin G (IgG) receptors expressed by neutrophils also contribute to aggravated immune responses in IBD. IgG-Fc gamma receptors (FcγRs) are associated with the pathogenesis of various IgG-dependent immune diseases, such as IBD, rheumatoid arthritis, systemic lupus erythematosus and systemic anaphylaxis.^57,58^ Neutrophils constitutively express FcγRs, which mainly encompass FcγR I (CD64), FcγR II (CD32) and FcγR III (CD16), to facilitate the phagocytosis of IgG-opsonized microbiota and drive cell activation via crosslinking of several receptors.^59^ Neutrophil expression of FcγR I, which is capable of recognizing and phagocytizing immune complexes, executing antibody-dependent cytotoxicity and triggering respiratory burst, is upregulated in adult patients with active IBD.^60,61^ Soluble FcγR IIIb (sFcγRIIIb) is also increased in both CD and UC patients, with the concentration of sFcγRIIIb reflecting the degree of mucosal inflammation.^62^ Additionally, monoclonal antibodies against TNF have been found engaged with FcγRs. The Fc-FcγR interaction is required for the therapeutic efficacy of anti-TNF in IBD. Consistently, activated FcγR-deprived mice fail to effectively response to anti-TNF therapy in T-cell transfer colitis model, while administration of anti-TNF monoclonal antibodies with enhanced Fc-binding affinity shows improved efficacy.^63^

### Neutrophils undermine the resolution of intestinal inflammation

Neutrophils are short-lived, spontaneously die in apoptosis after an average circulatory lifespan of approximately 3 hours for mouse cells and 16 hours for human.^64^ Previous study has reported that nearly 10^9^ peripheral neutrophils per kg of body weight are refurbished daily under physiological conditions.^65^ The engulfment process of apoptotic neutrophils performed by phagocytes such as macrophages, termed as efferocytosis, prevents secondary lysis and extravasation of noxious neutrophil granules, dampens pro-inflammatory signaling, and ameliorates immune responses, which appears to be crucial for maintenance of intestinal mucosal homeostasis and is a prerequisite for the resolution of intestinal inflammation.^46,66,67^ Moreover, apoptotic neutrophils exert anti-inflammatory effects and trigger pro-resolving responses in monocytes, macrophages and dendritic cells.^68^ As described previously, the longevity of neutrophils can be extended by pro-inflammatory mediators during intestinal inflammation of IBD, which allow neutrophils to carry out more durable activities and accumulate in the gut mucosa. Given that efferocytosis of apoptotic neutrophils is a fundamental process required for resolution of intestinal inflammation and maintenance of mucosal homeostasis, its dysregulation leads to over-activated immune responses, excessive inflammation, and uncontrollable infection. Thus, approaches that regulate and enhance efferocytosis can be harnessed to combat intestinal microbial infection and mucosal inflammatory damage.

Moreover, infiltrated neutrophils are able to utilize membrane-derived microparticles to deliver active inflammatory mediators (e.g. MPO, elastase and MMPs) to modulate local immune responses. Specifically, MPO, which is abundantly cumulated in neutrophil azurophilic granules used for pathogen killing, mobilizes to the neutrophil surface and subsequently releases within microparticles to promote neutrophil activation and binds to IECs, leading to potent inhibition of epithelial wound healing.^69^ Impeding epithelial migration and wound closure are observed in the colonic biopsy of a wound murine experiment model through inhibition of IEC proliferation (shown by Ki67 staining) *in vivo*. Another cytotoxic granule, neutrophil elastase (NE), released by infiltrating neutrophils *in situ* during inflammatory responses, is involved in mucosal damage and repairment in patients with UC. NE normally serves in mucosal defense against intruding microbial pathogens, but can also cause impediment of tissue repairment when produced in an excessive manner. In patients with UC, NE is observed to be cumulated in the inflamed mucosa and undermines mucosal restitution through suppressive effects on recombinant hepatocyte growth factor (rHGF)-induced IEC proliferation.^70^ Additionally, on reaching the intestinal epithelial surface, membrane-derived microparticles released by neutrophils deposit onto the apical epithelium. These microparticles containing miRNAs and MMP-9 elicit destruction of epithelial intercellular adhesions by cleaving desmoglein-2 (instead of E-cadherin) in a MMP-9-dependent manner *in vivo*, leading to elevated neutrophil infiltration and inflammation in the ligated intestinal loop murine model.^71^

These data elucidate the nonnegligible roles of neutrophils in amplifying immune responses and preventing resolution of intestinal mucosal inflammations, contributing to a persistent course of IBD.

### Neutrophils participate in the thrombosis in IBD

Patients with active IBD have an elevated incidence of microvascular thrombosis and thromboembolism which are associated with enhanced procoagulant phosphatidylserine (PS) exposure on platelets (PLTs) and increased platelet microparticles (MPs).^72,73^ Despite the pathogen-killing effects of NETs, an increase of NETs in plasma and the deposition in colon tissues are observed in active IBD, followed by exacerbated colon tissue damage and increased thrombotic tendency. NETs are also found to be accumulated in DSS-induced murine colitis model, and inhibition of NETs through DNase administration could attenuate mucosal inflammation as well as thrombus formation.^74^ Evidenced by experiment results, NETs have been demonstrated to activate PLTs, which then promote coagulation via externalizing PS and releasing procoagulant MPs *in vitro*. Consistently, active UC-derived NETs are found to convert human umbilical vein cells (HUVECs) to a procoagulant phenotype due to increased PS expression.^74^

### Dysfunctional neutrophils are associated with therapeutic failure in UC

Accumulated neutrophils are found in inflamed colon of UC patients with corticoid-resistance or cyclosporine A-unresponsiveness, accompanied with high levels of ROS, NE and uncontrolled T cell proliferation,^75,76^ indicating that increased neutrophil infiltration may be associated with the therapeutic failure in UC patients. The enrichment of twist-1 has been found in neutrophils isolated from inflamed colon of UC patients with corticoid-resistance, which interacts with glucocorticoid receptor α to attenuate the effects of steroids on regulating neutrophil activities. Moreover, the inhibition of twist-1 can block neutrophil-mediated corticoid-resistance in colitis mice and restore sensitivity to steroid therapy in the colon.^75^ Another therapeutic approach in the treatment of UC patients, cyclosporine A, can robustly induce effective clinical remission in severe steroid-refractory UC.^77^ However, the rate of short-term nonresponse to cyclosporine A is approximately 29%, and neutrophils are proved to be associated with the nullity of cyclosporine A therapy.^76,78^ Cyclosporine A is observed to suppress the migration and the apoptosis of neutrophils in response group, dampens release of IL-8, ROS and antimicrobial peptides in neutrophils, and transforms the neutrophil from a pro-activated status to a prolonged, pro-glycolytic and quiescent status, thereby driving the resolution of gut inflammation.^76^

### Neutrophil dysfunction contributes to intestinal mucosal inflammation

Although most studies have shown that neutrophils accumulate and overact in the intestinal mucosa of patients with IBD, there is considerable evidence demonstrating the association between defective neutrophil function and intestinal inflammation. For example, defective superoxide generation and phagocytosis are found in neutrophils from patients with IBD,^79,80^ which thus contribute to reduced clearance of intestinal pathogens and exacerbated lymphocyte-mediated immune response.^81^ Furthermore, neutrophil dysfunction appears to be associated with compromised macrophage properties including decreased production of pro-inflammatory cytokines (e.g. G­CSF, IL-6, IFN-γ and TNF-α) and neutrophil chemoattractants in CD, which further dampen neutrophil functions.^82,83^ Accumulated neutrophils at the sites of microbial infection show markedly impaired clearance of bacteria in CD. Impaired accumulation of neutrophils was observed in traumatized rectal mucosa from eight patients with CD (79% reduction, n = 8, *P* = 0.0003) compared to healthy individuals. Consistently, low frequency of IL-8-positive cells was observed in CD patients (63% reduction, n = 8, *P* = 0.003). Further, IL-8 production by macrophages from CD patients was significantly reduced (38% reduction, n = 50, *P* < 0.0001), together with decreased production of C5a (48% reduction, n = 41, *P* = 0.0005) and TNF-α (52% reduction, n = 27, *P* < 0.0001). Similar defects of neutrophil accumulation were also found in the impairment applied to the ileum (57% reduction, n = 3, *P* = 0.05 for neutrophils; 63% reduction, n = 3, *P *= 0.05 for IL-8-positive cells).^83^ These findings indicate that decreased neutrophil accumulation and IL-8 secretion at the sites of acute inflammation in the intestinal mucosa, indicative of abnormal neutrophil response, are associated with persistent tissue damage in CD. Moreover, intruding bacteria may remain in the mucosal tissues due to suboptimal destruction by neutrophils, and are then phagocytized by macrophages instead.

Taken together, accumulating lines of evidence highlight a pathogenic role of neutrophils in the destruction of epithelial barrier integrity, aberrant immune responses to microbiota, development of the intestinal inflammation, and therapeutic failure of IBD. It also manifests that both hyperactivation and functional deficiency of neutrophils contribute to pathological intestinal inflammation, which emphasizes the multiple roles of neutrophils in intestinal inflammatory diseases.

## Restorative roles of neutrophils in tissue repair of IBD

In addition to fueling inflammation response by ROS, cytotoxic intracellular granular contents (e.g. MPO, defensins, lysozyme, elastase, proteases and hydrolases), and NETs, neutrophils also have the healing potential to exert restorative actions including eliminating microbe translocation, facilitating angiogenesis and aiding in the resolution of mucosal wounds.

### Neutrophils eliminate microbial invasion

Contrary to previously referred animal experiments,^55,56^ neutrophil depletion *in vivo* aggravates both experimental acute colitis in mice induced by dinitrobenzene sulfonic acid (DNBS) or DSS and chronic colitis in severe combined immunodeficient mice reconstituted with CD4^+^CD45RB^high^ T cells, and enhances translocation of microbes in colitic mice, indicative of a beneficial role of neutrophils during the intestinal inflammation.^84–86^ The characteristics of rapid mobilization and chemoattract endow neutrophils with profound bactericidal capacity after effective recognition and capture of invading microbes. Especially, a unique subset of neutrophils, CD177^+^ neutrophils, are capable of producing higher levels of IL-22 and transforming growth factor (TGF)-β to promote tissue healing, and dampening the production of pro-inflammatory cytokines (e.g. IL-6, IL-17A and IFN-γ) to restrain the inflammatory responses compared with CD177^−^ neutrophils. Moreover, functionally activated CD177^+^ neutrophils exhibit increased bactericidal activities (e.g. ROS, antimicrobial peptides and NETs) which demonstrate its protective role in modulating intestinal mucosal inflammation.^87^ Dysfunctional neutrophils in the intestinal mucosa under massive bacterial invasion promote the susceptibility to disease, suggesting a dominant role of the requirement of neutrophils in preventing bacterial reproduction in gut mucosa.^88^ The intestinal inflammation in IBD patients is characterized by mucosal damage, increased epithelial permeability, intrusion of commensal microbiota into the subepithelial space or lamina propria and exceeded infiltration of neutrophils. Removal of dead cells and microbiota by neutrophil phagocytic activity contributes to clear the area for mucosal barrier remodeling which is a prerequisite for the resolution of the intestinal inflammation.^89^ Collectively, these data indicate that neutrophils play a key role in manipulating intestinal bacterial homeostasis and regulating the inflammatory response, which are tightly modulated by limiting excess translocation of commensal microbes and fighting against invasion by bacterial pathogens under conditions of inflammatory diseases.

### Neutrophils promote angiogenesis in gut mucosa

Angiogenesis mediated by multiple factors is fundamental to restoration of damaged epithelia in IBD. Vascular endothelial growth factor A (VEGF-A) as one of major proangiogenic factors triggers angiogenic activities of human intestinal microvascular endothelial cells (HIMECs) via VEGF receptor 2 (VEGFR-2) *in vitro* and facilitates neutrophil adherence to intestinal endothelium through intercellular adhesion molecule-1 (ICAM-1) *in vivo*.^90^ Furthermore, it is reported that CD11b^+^/Gr-1^+^CXCR4^high^ neutrophils, a proangiogenic circulating neutrophil subset, are recruited by VEGF-A and CXCL-12 and release additional effector protein MMP-9 to augment the initiation of angiogenesis after inflammatory tissue damage.^91–93^ Noteworthily, only neutrophil-derived MMP-9 at metalloproteinases (TIMP)-free status owns proangiogenic capacity and serves as unique proangiogenic molecule at the sites of inflammation.^93^ These findings prove that infiltrated neutrophils deliver potent proangiogenic mediators and promote angiogenesis during intestinal inflammation.

### Neutrophils promote tissue restoration

One of primary effects of neutrophils is eliminating microbial intrusion at the sites of mucosal injuries, but they also function to restore damaged tissue during wound healing. Specific pathological characteristic of IBD is the immoderate inflammatory responses followed by the persistence of mucosal epithelial injuries. At the sites of injuries, neutrophils initiate healing program via direct effects not only on angiogenesis but also on cell proliferation. During the acute inflammatory phase, neutrophils play a critical role in cleansing damaged tissue by eliminating exogenous bacterial pathogens, and contributing to the production of growth factors, such as VEGF-A,^94^ and lipid mediators, including resolvins, lipoxins and protectins, which promote wound healing.^95–97^ Moreover, accumulated neutrophils bind to the apical epithelium through the interaction with increased expression of ICAM-1 under inflammatory conditions, which results in reduced neutrophil apoptosis, increased IEC proliferation and accelerated wound healing via Akt and β-catenin signaling.^98^

The functions of neutrophils, including bactericidal actions, angiogenesis and tissue restoration, are tightly relevant to healing mucosal wounds secondary to aberrant inflammatory responses and the restoration of intestinal homeostasis.

## Regulation of neutrophil functions and the therapeutic prospects in IBD

It is well accepted that neutrophils exert potent effects on recruitment and activation of crucial immune cells involved in intestinal inflammation. On the other hand, several cytokines and chemokines produced by IECs and other immune cell types during intestinal inflammation also tightly regulate neutrophil migration and functions. For example, IL-8, an effective chemoattractant of neutrophils produced by IECs, promotes infiltration of neutrophils from lamina propria to epithelium.^21^ Moreover, classic neutrophil chemoattractants (e.g. CXCL-1, CXCL-8, CXCL-10, GM-CSF, G-CSF, IL-1β, IL-6 and TNF-α) produced by different cell types, such as macrophages, IECs and fibroblasts, have been shown to contribute to neutrophil recruitment and activation. However, non-classical chemoattractants of neutrophils such as growth-regulated oncogene-α (GRO-α) have also been demonstrated to be essential for neutrophil recruitment during intestinal inflammation.^99^

As highlighted above, neutrophils comprise a vital component of innate immune system and are equipped with sufficient bactericidal weapons to defend against invading microbiota. However, microbial ingredients such as lipopoproteins, lipopolysaccharide, and various metabolites, also have potent effects on neutrophils and other multiple intestinal cell types (e.g. IECs, Paneth cells, goblet cells, enteroendocrine cells, myofibroblasts, macrophages, monocytes, dendritic cells and T cells) and subsequently trigger a multitude of Toll and Nod-like receptor-mediated responses, participating in both the maintenance of intestinal mucosal homeostasis and development of pathological intestinal inflammation.^100,101^ Bacteria also produce metabolites capable of directly recruiting and activating neutrophils, such as fMLF and short chain fatty acids (SCFAs). The peptide fMLF owns potent chemoattractant effect on neutrophils, and increased expression of its receptor (i.e. N-formyl peptide receptor) is observed in CD patients.^35^ Microbial metabolites are capable of modulating intestinal immune responses and homeostasis. Microbial metabolites butyrate belongs to SCFAs and inhibits activated neutrophils isolated from IBD patients to produce pro-inflammatory cytokines, ROS, MPO and calprotectin in a histone deacetylase inhibitors (HDACi)-dependent manner. Butyrate also suppresses the formation of NETs and migration of neutrophils isolated from both CD and UC patients *in vitro*. Consistently, *in vivo* experiments also demonstrate that oral administration of butyrate alleviates mucosal inflammation in DSS-induced colitic mice through restricting neutrophil-associated immune responses such as production of pro-inflammatory cytokines and NET formation.^102^

Taken together, these findings highlight crucial roles for inflammatory mediators from other cell types and microbiota-derived products in manipulating neutrophil infiltration and function during intestinal inflammation through inflammatory mediators such as cytokines and chemokines, indicative of synergistic roles of various cell types in modulating neutrophil properties to further affect the onset and resolution of intestinal inflammation in IBD. Thus, combined with the previously described facts that neutrophil dysfunction is involved in the therapeutic failure of UC, approaches to regulate neutrophil dynamics (e.g. migration and infiltration towards the inflamed mucosa) and functions (e.g. release of ROS, phagocytosis, degranulation and formation of NETs) may provide a novel approach for treatment of IBD.

## Conclusions and future perspectives

**Figure 1.**
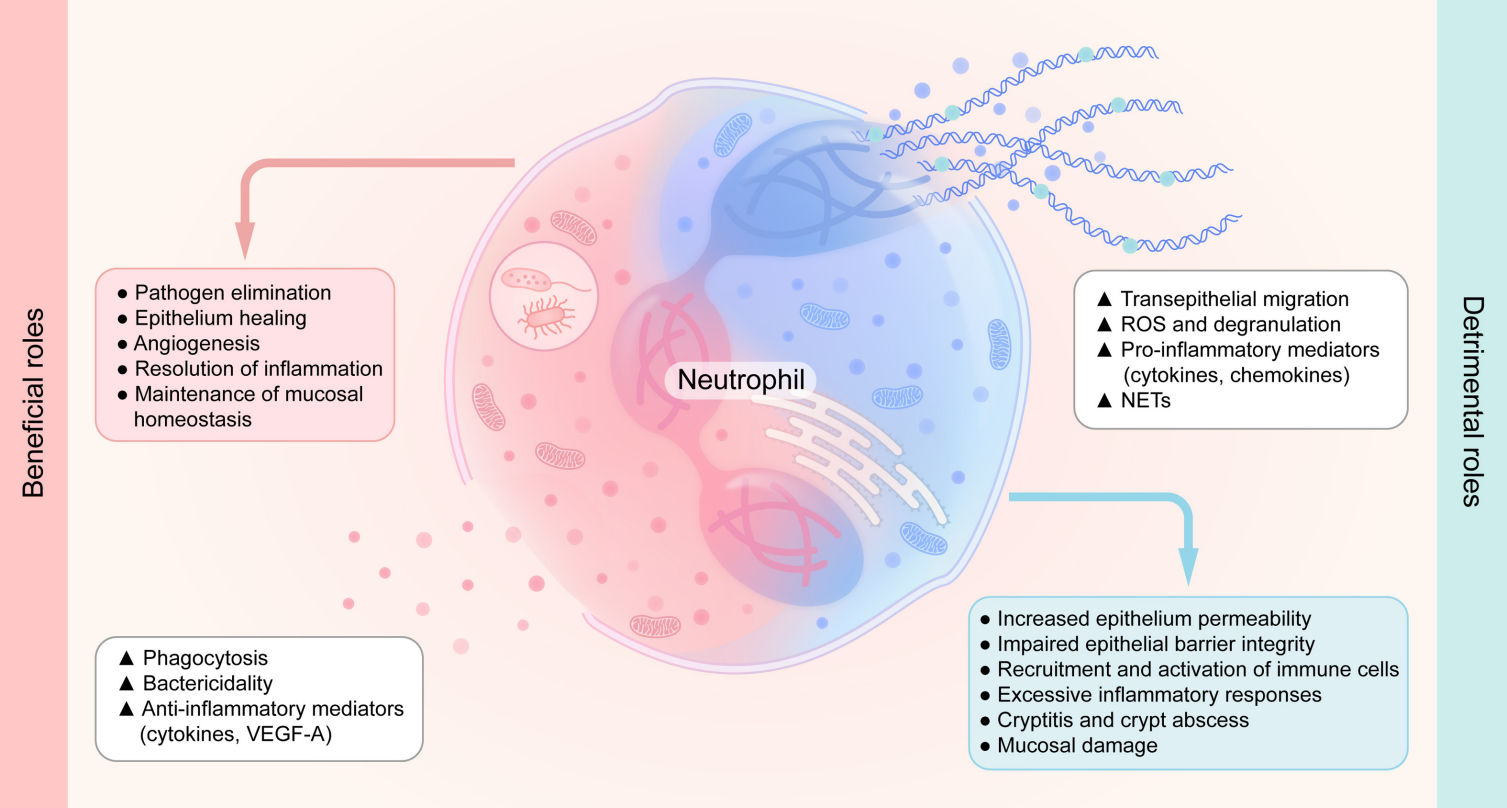
The ‘yin’ and ‘yang’ of neutrophil functions in the pathogenesis of IBD. Neutrophils not only participate in the mucosal injury, aberrant responses to icrobiota, extensive intestinal inflammation, and therapeutic failure in IBD, but also play an important role in the elimination of pathogens, angiogenesis and wound healing, which is a prerequisite for the restoration of intestinal inflammation. All this knowledge elucidates the beneficial and deleterious roles of neutrophils in the pathogenesis of IBD.

Although the understanding of immune regulation and immune response of neutrophils has gained great improvement during gut inflammation, especially in human IBD, the beneficial and detrimental roles of neutrophils remain controversial. Indeed, neutrophils are critical for the maintenance of mucosal homeostasis. It is also well known that neutrophils act as a double-edged sword by not only contributing to the restoration of mucosal inflammatory damage through the clearance of detrimental pathogens and the promotion of mucosal wound healing, but also participating in the excessive immune response and the extensive intestinal mucosal inflammation, owing to the release of toxic granules and massive transepithelial infiltration (Fig. 1). The contradictory ‘yin’ and ‘yang’ of neutrophil functions in the pathogenesis of IBD are manipulated by complex parameters involved in the migration, activation, immunoregulatory functions and apoptosis processes. Thus, better understanding of neutrophil properties in IBD will be crucial to uncover potential targets exploited in disease etiology and provide new insights for orchestrating neutrophil functions to counterbalance intestinal inflammation.
